# Visceral leishmaniasis outbreaks in Bihar: community-level investigations in the context of elimination of kala-azar as a public health problem

**DOI:** 10.1186/s13071-020-04551-y

**Published:** 2021-01-15

**Authors:** Khushbu Priyamvada, Joy Bindroo, Madan Prashad Sharma, Lloyd A. C. Chapman, Pushkar Dubey, Tanmay Mahapatra, Allen W. Hightower, Caryn Bern, Sridhar Srikantiah

**Affiliations:** 1 CARE-India Solutions for Sustainable Development, Patna, India; 2 Bihar State Programme (Kala-azar), Patna, India; 3 Centre for Mathematical Modelling of Infectious Diseases, London School of Hygiene and Tropical Medicine, London, UK; 4 Independent consultant, Bangkok, Thailand; 5 Department of Epidemiology and Biostatistics, University of California San Francisco, San Francisco, CA USA

**Keywords:** Visceral leishmaniasis, Epidemiology, Risk factors, Outbreak investigation, India

## Abstract

**Background:**

With visceral leishmaniasis (VL) incidence at its lowest level since the 1960s, increasing attention has turned to early detection and investigation of outbreaks.

**Methods:**

Outbreak investigations were triggered by recognition of case clusters in the VL surveillance system established for the elimination program. Investigations included ascertainment of all VL cases by date of fever onset, household mapping and structured collection of risk factor data.

**Results:**

VL outbreaks were investigated in 13 villages in 10 blocks of 7 districts. Data were collected for 20,670 individuals, of whom 272 were diagnosed with VL between 2012 and 2019. Risk was significantly higher among 10–19 year-olds and adults 35 or older compared to children younger than 10 years. Outbreak confirmation triggered vector control activities and heightened surveillance. VL cases strongly clustered in tolas (hamlets within villages) in which > 66% of residents self-identified as scheduled caste or scheduled tribe (SC/ST); 79.8% of VL cases occurred in SC/ST tolas whereas only 24.2% of the population resided in them. Other significant risk factors included being an unskilled non-agricultural laborer, migration for work in a brick kiln, living in a kuccha (mud brick) house, household crowding, habitually sleeping outside or on the ground, and open defecation.

**Conclusions:**

Our data highlight the importance of sensitive surveillance with triggers for case cluster detection and rapid, careful outbreak investigations to better respond to ongoing and new transmission. The strong association with SC/ST tolas suggests that efforts should focus on enhanced surveillance in these disadvantaged communities.

**Graphical Abstract:**

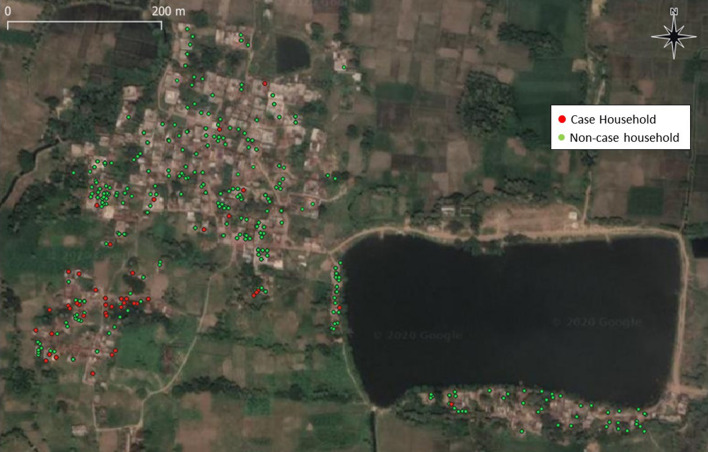

## Introduction

In the Indian subcontinent, visceral leishmaniasis (VL) is caused by the protozoan parasite *Leishmania donovani* and transmitted by the sand fly *Phlebotomus argentipes* [1]. The only proven reservoir is human, and the most severe form of the disease is called kala-azar (black fever). Female sand flies become infected when they take bloodmeals from patients with kala-azar or those with post-kala-azar dermal leishmaniasis (PKDL), a dermatosis that follows VL in 5 to 15% of apparently cured VL patients [2, 3].

In India, suppression of VL incidence during the malaria eradication campaigns of the 1950s–1960s was followed by three large-scale epidemic waves, peaking in 1978, 1992 and 2007 [4]. Throughout this time, Bihar has been the most severely affected state in India, with endemic districts concentrated north of the Ganges River. A regional effort by the governments of India, Bangladesh and Nepal, coordinated by the World Health Organization (WHO), aims to eliminate VL as a public health problem (defined as VL incidence < 1 per 10,000 population at the subdistrict level) [5]. The main program components comprise rapid VL diagnosis followed by effective treatment, vector control and systematic disease surveillance [6]. CARE-India conducted a VL situation assessment in 2013 [7], followed by the refinement and establishment of the online Kala-azar Management Information System (KAMIS) to provide ongoing surveillance under the auspices of the National Vector-Borne Disease Control Programme (NVBDCP).

Visceral leishmaniasis incidence in the Indian subcontinent is now at its lowest level since the 1960s [8]. In this setting of low incidence, increasing attention has turned to early detection of outbreaks, defined by WHO/NVBDCP as a newly reported VL or PKDL case in a village with no reported cases for at least 3 years; a cluster of three or more VL or PKDL cases reported in a village within 4 weeks; or an increase in case numbers in an endemic block over an unspecified outbreak threshold [9]. Outbreak investigations consist of a series of steps to confirm the outbreak and define its extent, initiate appropriate interventions, and when feasible, determine risk factors for disease [10]. We conducted a series of community-level VL outbreak investigations from 2017 to 2019. Our aims were to understand the epidemiology of VL clusters meeting the VL outbreak definition and to evaluate risk factors for VL within the outbreak communities. In coming years, prevention of VL resurgence will depend upon the ability of the public health system to anticipate and detect such outbreaks and to rapidly institute effective control measures.

## Methods

Two of our investigations were conducted in blocks that had mean annual VL incidence below the elimination threshold of 1 per 10,000 population from 2009 to 2011; we considered these newly affected blocks. The other investigations occurred in eight known endemic blocks whose mean incidence was > 2 per 10,000 from 2009 to 2011. The first investigation was conducted in 2017 in Kosra village, Sheikhpura block, where a large cluster of newly recognized VL cases received considerable press coverage and attention from public health authorities [11, 12]. Subsequent to this investigation, kala-azar block coordinators (KBCs) deployed by CARE throughout Bihar to assist VL elimination program operations were asked to report unexpected VL case clusters, and KAMIS data were evaluated on a regular basis to identify potential outbreaks. In all investigated villages, the field team worked with local public health officials to confirm and map VL cases and to ensure that control program interventions were instituted. We conducted a full census of each village and collected data on all residents to facilitate the risk factor assessment. Data included demographics, social status, house construction materials, migration history, occupation, sleeping practices and domestic animal ownership.

Kala-azar block coordinators report VL cases in KAMIS after confirmation at the primary health center based on the nationally mandated case definition (at least 2 weeks of fever, splenomegaly and positive rK39 rapid test or biopsy) [6]. Report date reflects the date of confirmation. As part of the ongoing surveillance system, KBCs review medical records for each VL patient and collect case details using a structured data format. The collected data include determination of illness duration prior to treatment using a calendar based on locally memorable events such as festivals. Our epidemiological analyses were based on the illness onset month determined in this way.

### Statistical analysis

Epidemiological curves were constructed for each village or geographical cluster of villages, based on month and year of fever onset. To provide a visual assessment of geographic clustering, case and non-case households were mapped based on GPS readings. Potential risk factors for VL were evaluated in log-binomial regression models, with generalized estimating equations to account for intra-household correlation. Multivariable models were constructed using a stepwise selection procedure with significance level of 0.05. Analysis was performed in SAS version 9.4.

## Results

Between December 2017 and April 2019, VL outbreaks were identified and investigated in 13 villages in 10 blocks of 7 districts (Table 1) (see also Additional file 1: Figure S1). Two blocks south of the Ganges River, Sheikhpura and Nawada, had mean annual VL incidence < 1 per 10,000 in 2009–2011. The other eight blocks, all north of the Ganges River, had mean annual incidence between 2 and 7 per 10,000 population during 2009–2011. VL surveillance data are not available at the block level prior to 2009. Data were collected for 20,670 individuals in these 13 villages, of whom 272 were diagnosed with VL between 2012 and 2019 (Table 2).

**Table 1:** Locations in which visceral leishmaniasis (VL) outbreak investigations were conducted, Bihar, 2017–2019

District	Block	VL incidence, 2009–2011^a^	Villages investigated	Village population	
				Total *N*	VL cases^b^
Endemic blocks			Villages in endemic blocks		
Muzaffarpur	Paroo	6.19	Pandey	3258	11
Purnia	Banmanhki	6.76	Binowagram	2971	34
Purnia	Bhawanipur	4.29	Bhamath	1379	3
Purnia	Krityanand Nagar	6.36	Pansohi	209	9
Saharsa	Mahishi	6.65	Thanwar	1757	4
Saharsa	Nauhatta	4.25	Rasalpur	2380	15
Saran	Dariyapur	2.77	Jitwarpur	3044	25
Sitamarhi	Dumra	2.01	Chakka Majhauliya, Chakka Rasalpur, Panapur	545	31
Newly affected blocks			Villages in newly affected blocks		
Nawada	Kashichak	0.05	Kashichak, Lal Bigha	2692	66
Sheikhpura	Sheikhpura	0.90	Kosra	2435	74

^a^Mean annual VL incidence in cases per 10,000 population from 2009 to 2011. Data prior to 2009 not available at the block level

^b^Cumulative number of VL cases in the study population, 2012 to 2019

**Table 2:** Study population characteristics, visceral leishmaniasis (VL) outbreak investigations conducted in Bihar 2017–2019

Characteristic	Population^a^		VL^b^		PKDL^c^	
	*N*	%	*N*	%	*N*	%
Sex^d^						
Male	10693	51.8	165	60.7	3	37.5
Female	9964	48.2	107	39.3	5	62.5
Age group^e^						
< 10	5297	25.7	55	20.2	2	25.0
10–19	4753	23.0	79	29.0	2	25.0
20–34	4828	23.4	52	19.1	2	25.0
35–49	3060	14.8	44	16.2	1	12.5
≥ 50	2699	13.1	42	15.4	1	12.5
Lives in SC/ST tola^f^						
Yes	5006	24.2	217	79.8	4	50.0
No	15664	75.8	55	20.2	4	50.0

^a^Total *N* = 20,670

^b^Four subjects with VL prior to 2012 excluded from VL analyses

^c^Includes four VL patients with onset in 2012 (3) and 2014 (1) who are also listed in VL column. Four others with onset prior to 2012 are not included in VL column

^d^Sex data missing for 13 individuals

^e^Age data missing for 33 individuals

^f^SC/ST, Scheduled caste/scheduled tribe; tola classification presented in detail in Additional file 2: Table S1

**Fig. 1:**
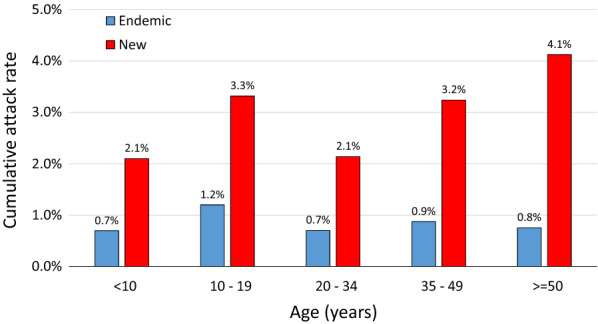
Age-specific patterns of visceral leishmaniasis incidence in communities located in historically endemic blocks compared to newly affected blocks. The cumulative incidence in communities in endemic blocks was highest in the 10–19 year age group and low in older age groups. In blocks with newly recognized transmission, the cumulative incidence was substantially higher than in endemic blocks and was highest in the oldest age group

All age groups were affected by VL, but risk was significantly higher among 10–19 year-olds and adults 35 years or older compared to children younger than 10 years (Table 3). In univariable analyses, adult males had significantly higher risk than adult females, but the differences by sex within age groups did not reach statistical significance, and there was no significant interaction between age and sex (*p* = 0.49 by Breslow-Day test for interaction). Age-specific patterns of VL incidence differed markedly in communities located in historically endemic blocks compared to newly affected blocks (Fig. 1). The cumulative incidence by age group in communities in endemic blocks ranged from 0.7 to 1.2%, with a peak in the 10–19 year age group and low incidence in older adults. In blocks with newly recognized transmission, the cumulative incidence was substantially higher (2.1 to 4.1%) than in endemic blocks and was highest in the oldest age group.

**Table 3:** Univariable analyses of risk factors for visceral leishmaniasis (VL) based on log-binomial regression models

Characteristic	Cumulative VL incidence	Univariable analyses		
	*n*/*N* (%)	Relative risk	95% CI	*p*
Sex				
Male	165/10689 (1.5)	1.42	1.15, 1.76	0.0009
Female	107/9960 (1.1)			
Age group (years)				
< 10	55/5296 (1.0)	Referent		
10–19	79/4751 (1.7)	1.69	1.18, 2.43	0.005
20–34	52/4827 (1.1)	1.20	0.84, 1.72	0.312
35–49	44/3059 (1.4)	1.54	1.06, 2.26	0.025
≥ 50	42/2696 (1.6)	1.71	1.19, 2.47	0.004
Lives in predominantly SC/ST tola^a^				
Yes	217/5004 (4.3)	12.37	8.79, 17.41	< 0.0001
No	55/15658 (0.4)			
Unskilled non-agricultural laborer				
Yes	73/1143 (6.4)	3.95	2.49, 6.26	< 0.0001
No	199/19519 (1.0)			
Ever sleeps outside				
Yes	166/7528 (2.2)	2.40	1.83, 3.14	< 0.0001
No	106/13106 (0.8)			
Sleeps on ground				
Yes	155/3918 (4.0)	4.43	3.22, 6.10	< 0.0001
No	117/16686 (0.7)			
Sleeps under net^b^				
Ever	145/15764 (0.9)	0.49	0.31, 0.80	0.004
Never	52/2462 (2.1)			
Sleeps under net^b^				
Always	71/10963 (0.7)	0.47	0.32, 0.71	0.0003
Sometimes	74/4801 (1.5)	0.73	0.43, 1.24	0.246
Never	52/2462 (2.1)	Referent		
Defecation site				
Field	245/13204 (1.9)	5.28	3.28, 8.49	< 0.0001
Latrine or toilet	27/7451 (0.4)			
Caste				
Scheduled caste/tribe	219/6310 (3.5)	9.40	6.52, 13.53	< 0.0001
Other/general	53/1432 (0.4)			
House materials^c^				
Kuccha	174/9560 (1.8)	2.00	1.42, 2.79	< 0.0001
Semipucca or pucca	98/11086 (0.9)			
Crowding				
≥ 3 people per room	186/10675 (1.7)	1.95	1.40, 2.73	< 0.0001
< 3 people per room	86/9968 (0.9)			
Owns cow				
Yes	109/11317 (1.0)	0.58	0.41, 0.80	0.0007
No	163/9336 (1.8)			
Owns goats				
Yes	132/8436 (1.6)	1.39	1.00, 1.93	0.047
No	140/12217 (1.2)			
Owns pigs				
Yes	21/456 (4.6)	3.39	1.88, 6.12	< 0.0001
No	251/20197 (1.2)			
Migrated for work in past 3 years				
Yes	96/4472 (2.2)	1.47	1.11, 1.94	0.007
No	176/16190 (1.1)			
Migrated to work in brick kiln				
Yes	75/1053 (7.1)	5.06	3.30, 7.76	< 0.0001
No	197/19609 (1.0)			

Generalized estimating equations used to account for intra-household correlation

^a^SC/ST, Scheduled caste/scheduled tribe; tola classification presented in detail in Additional file 2: Table S1

^b^Data missing for 2444 respondents, including 75 VL cases

^c^*Kuccha*, unfired mud brick; *pucca*, cement or fired brick; semi-*pucca*, a mixture of the two

**Fig. 2:**
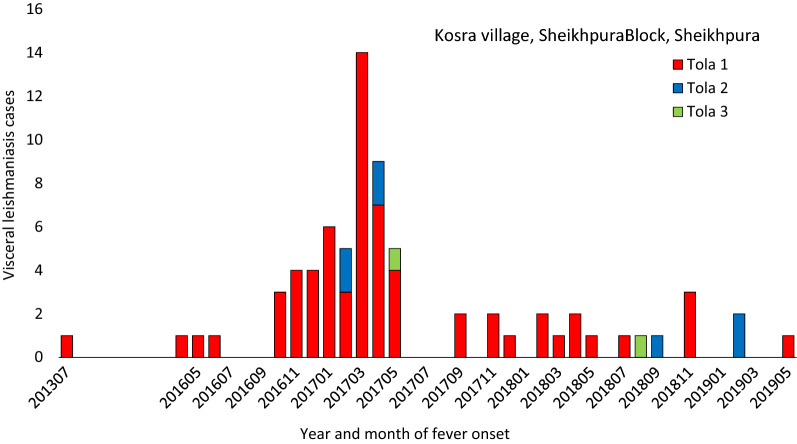
Visceral leishmaniasis case count by year and month of fever onset in Kosra village, Sheikhpura block, Sheikhpura district. The first recognized patient-reported fever onset in 2013 but was not treated until late 2014. Interventions, including indoor residual spraying, first occurred in March 2017, but cases continued to be reported in 2018 and 2019

**Fig. 3:**
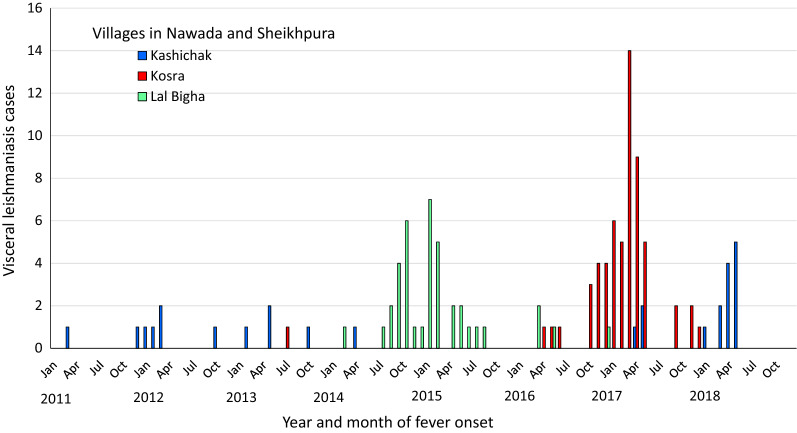
Visceral leishmaniasis case count by year and month of fever onset in Kosra village in Sheikhpura district and Lal Bigha and Kashichak villages in Nawada district. These blocks are located south of the Ganges River and were not considered endemic for VL. Social links between villages may have facilitated low-level sustained transmission prior to recognition of the large outbreak in Kosra in 2017

**Fig. 4:**
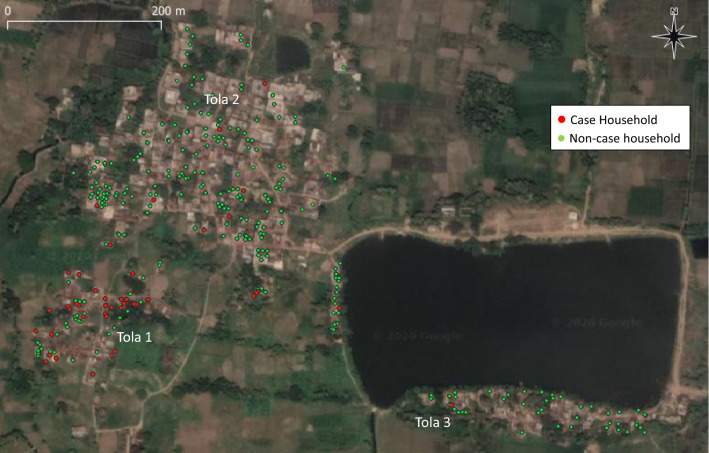
Map of houses with (red) and without (green) visceral leishmaniasis cases from 2013 to 2019 in Kosra village, Sheikhpura block, Sheikhpura district. In Tola 1, 44% of households had at least one case

Epidemiological curves reveal that VL cases occurred in outbreak villages over a period of several years (Figs. 2 and 3) (see also Additional file 1: Figures S2–S5). South of the Ganges, three nearby villages had VL cases occurring at a low rate from 2013 onward, with increasing transmission leading to larger clusters in Lal Bigha in 2015 and Kosra in 2016–2017 (Fig. 3). Case maps indicate spatial clustering within villages, often within a single tola (hamlet within a village) (Fig. 4) (see also Additional file 1: Figures S6–S8). In all villages, confirmation of the outbreak triggered vector control activities and heightened surveillance through active case detection that continued for at least 1 year following the last detected VL case.

An array of factors, most with evident links to poverty, were associated with a significant increase in VL risk (Table 3). The strongest determinant was residence in a tola in which two-thirds or more of the residents self-identified as scheduled caste or scheduled tribe (SC/ST), associated with a relative risk of 12.4 (95% confidence interval (CI) 8.8, 17.4). VL cases strongly clustered in these tolas; only 24.2% (5006/20,670) of the study population resided in SC/ST tolas, but 79.8% (217/272) of VL cases occurred within them (Additional file 2: Table S1). Self-identification as SC/ST was associated with a ninefold increase in risk (95% CI 6.5, 13.5), but the individual-level variable was less strongly predictive of VL than residence location.

Other significant risk factors included being an unskilled non-agricultural laborer, migration for work in a brick kiln, living in a kuccha (mud brick) house, household crowding, habitually sleeping outside or on the ground, and open defecation (Table 3). Sleeping under a bed net was associated with significant protection in the univariable analysis, with a trend toward a dose-response relationship. Unfortunately, net use data were missing for the villages investigated early in the process, which decreased statistical power for these analyses. Ownership of a cow was associated with significantly decreased risk. Ownership of a pig, while rare, was associated with significantly increased risk; ownership of goats was associated with increased risk that just reached statistical significance. We included questions on indoor residual insecticide spraying (IRS), but spraying occurred after illness onset for 84% of VL cases. Vector control activities occurred in response to the outbreaks and were not relevant as a predictor of disease risk. In the multivariable model, residence in an SC/ST tola remained the strongest predictor of disease, associated with a sevenfold increase in risk (95% CI 5.0, 10.6) compared to residence in other tolas (Table 4). Male sex, sleeping outside or on the ground, open defecation and migration to work in a brick kiln remained in the model with lower relative risk levels.

**Table 4:** Multivariable log-binomial regression model of risk factors associated with visceral leishmaniasis

Characteristic	Adjusted relative risk	95% confidence interval	*P*
Male sex	1.41	1.13, 1.77	0.003
Age group			
< 10	Referent		
10–19	1.84	1.27, 2.66	0.002
20–49	1.38	0.99, 1.92	0.06
≥ 50	1.94	1.30, 2.90	0.001
Lives in predominantly SC/ST tola^a^	7.29	5.03, 10.57	< 0.0001
Ever sleeps outside	1.54	1.12, 2.11	0.008
Sleeps on ground	1.61	1.14, 2.28	0.007
Open defecation	2.06	1.29, 3.30	0.003
Migrates to work in brick kiln	1.71	1.19, 2.46	0.004

The analysis dataset included 272 participants with and 20,357 participants without the disease. Model constructed using a stepwise selection procedure with significance level of 0.05, using generalized estimating equations to account for intra-household correlation

^a^SC/ST, Scheduled caste/scheduled tribe; tola classification presented in detail in Additional file 2: Table S1

In Kosra, where the biggest outbreak occurred, VL case occurrence was highly clustered in a single tola (Sheikhpura tola 1; Fig. 4). In tola 1, the cumulative incidence was 12.6% (65/516) compared to 0.4% (2/459) and 0.5% (7/1460) in tolas 2 and 3. Of 76 households in tola 1, 44% (34/76) had at least one case; 17 (22%) households had more than one member with VL. By contrast, a single VL case occurred in 3.1% (2/65) and 3.7% (7/187) of households in tolas 2 and 3, respectively. Although IRS was instituted soon after the outbreak was reported in May 2017, VL cases continued to occur, including 12 cases in 2018 and 3 cases in 2019 (Fig. 2).

The first recognized VL patient in Kosra had symptom onset in 2013 and was ill for 440 days before treatment in the private sector in 2015; this case was never reported to KAMIS (Fig. 2). The second VL patient in Kosra was the uncle of the first; he had onset in May 2016. Symptom duration was extremely prolonged in the early phase of the outbreak and shortened progressively after the outbreak was recognized and control measures instituted (median [IQR] days of illness 142 [115, 177], 55.5 [39, 69.5] and 20 [15, 30] for patients with onset in 2016, 2017 and 2018–2019, respectively). The mean patient age also decreased over the course of the outbreak (from 38.2 years in 2016 to 26.5 in 2017 and 20.3 in 2018/2019).

In Kosra, residence in tola 1 was associated with a relative risk of 28.2 (95% CI 14.0, 57.0); no other risk factors remained in an age-adjusted multivariable model. The strength of the tola 1 association was not due solely to poverty. Tola 2 was also SC/ST predominant and had higher rates of some poverty-associated risk factors than tola 1, including migration to work in a brick kiln (85% *vs* 46%; *p *< 0.0001), sleeping outside (59% *vs* 52%; *p *< 0.05) and sleeping on the ground (76% *vs* 71%; *p *> 0.05).

## Discussion

Despite remarkable progress toward the elimination of VL as a public health problem in India, transmission continues and VL outbreaks are increasingly recognized [13]. Our data suggest two different epidemiological scenarios. The age patterns observed in the villages north of the Ganges are characteristic of an endemic pattern. Herd immunity built up during high incidence periods causes a subsequent fall in case numbers, but after a hiatus of 5–10 years, VL returns predominantly in younger age groups not exposed in the earlier wave [4]. By contrast, in the villages south of the Ganges, where transmission was absent or very low during the last epidemic cycle, the highest VL incidence was in adults over 50. Interestingly, the very youngest age group appeared to be spared in both epidemiological patterns in the Indian subcontinent; this may be related to lower exposure due to different sleeping habits or other behavioral patterns.

Despite these differences, the salient risk factors were similar throughout our data and echo epidemiological studies conducted over the past 20 years [14–17]. Neighborhood-level clustering results from proximity to previous VL cases, the proven human infection reservoir; such spatial clustering is a well-recognized feature of VL [15, 18–22]. The occurrence of clusters in SC/ST tolas has been seen in other investigations in Bihar [19], and the intense local risk reflects a matrix of factors linked to poverty [23]. The risk is not due to caste *per se*. As seen in our data, not all SC/ST tolas are associated with risk, and an early study in the Nepali lowlands bordering Bihar showed no significant association with caste [16]. However, once an infectious VL case is present, closely situated houses of vulnerable construction, intra-household crowding, environmental factors that increase local sand fly density and behaviors that facilitate human-sand fly contact all come into play to promote transmission [17].

Epidemiological data, including the current analysis, support the century-old conclusion that kala-azar patients comprise the primary infection reservoir for *L. donovani* in India [24]. The question of whether there may be a secondary non-human reservoir host has come up repeatedly, but a definitive answer requires demonstration of infectiousness to sand flies and documentation of a sufficient population of infectious animals in settings of human VL incidence [25]. While household ownership of goats and pigs showed associations with increased risk in univariable models, neither variable remained in the multivariable model, suggesting they may be indirect indicators rather than directly associated with risk of disease. We did not collect data on dogs, the reservoir of *L. infantum* is other parts of the world; while stray dogs are common in Bihari villages, they are not connected to individual houses and roam freely, making it impossible to connect them to risk at the household level. The only study of dogs conducted in Bihar showed negative results by serology and PCR [26].

The protective association with cattle has been previously reported, though the effect of their presence is variable [15, 17, 27]. The presence of a cow may be associated with better nutritional status, which decreases the likelihood of an infection progressing to kala-azar [18, 28]. Cattle are an attractive bloodmeal source and may thereby increase local sand fly density. However, cattle may also divert flies away from humans, decreasing both biting and the prevalence of leishmanial infection in vectors. In a study in a highly endemic Bangladeshi village, the density of cattle around the individual’s house showed a strong protective association, whereas the association for ownership was substantially weaker, supporting the role of cattle in diverting sand flies away from humans, regardless of ownership [15].

A crucial unanswered question is where people become infected and how best to approach vector control. Although other sand fly species, including *P. papatasi* and *Sergentomyia spp*, are present in Bihar, *P. argentipes* has been established for many decades as the sole vector of *L. donovani* in northern India [29]. Because sand flies are active from dusk to dawn, with peak activity for *P. argentipes* recorded around midnight [30], sleeping patterns have long been assumed to alter risk [16]. Bed net use was protective in our univariable analyses, despite having data for only a portion of our study population; this finding is consistent with the protective association for both treated and untreated net use in observational data from Bangladesh and Nepal [15, 16, 31]. Nevertheless, a cluster-randomized trial of treated nets in India and Nepal failed to demonstrate protection against leishmanial infection, leading to the abandonment of nets as a major tool in the Indian VL elimination program [32]. Vector control in the Indian program relies almost exclusively on IRS, which has a direct impact only on sand flies resting inside houses [6]. Until 2015, the IRS program utilized dichlorodiphenyltrichloroethane (DDT) despite mounting evidence of resistance [33–35]; the program shifted to synthetic pyrethroids in 2015–2016. Recent data suggest that *P. argentipes* may be less endophilic than previously assumed [36], with trap yields in outdoor vegetation higher than those inside human dwellings, though lower than in mixed cow-human dwellings [37]. Vector surveillance has failed to show a consistent decrease in sand fly densities inside sprayed vs unsprayed houses [38], and VL cases continue to be reported to KAMIS from sprayed villages. Sleeping outdoors was a strong risk factor in the current analysis, suggesting outdoor transmission unlikely to be controlled by IRS. The risk associated with open defecation, which remained significant in the full multivariable model, is intriguing; perhaps vegetation stands constitute a location where humans and sand flies come into contact in the early morning hours [39].

Our data highlight the importance of sensitive surveillance with triggers for case cluster detection and rapid, careful outbreak investigations to better respond to ongoing and new transmission [8, 36]. The intense efforts to control the epidemic waves of the 1970s and 1990s were not followed by comprehensive VL surveillance, and the resurgence in the early 2000s caught the subcontinent unprepared. Without close local attention, VL transmission can pass unrecognized for several years, as occurred in Kosra. The outbreak there was amplified by the long symptomatic (and likely highly infectious) periods of the early VL cases and lack of pre-existing population immunity. No other case cluster we investigated had an attack rate approaching that in Kosra.

## Conclusions

In the future, as overall VL incidence falls even farther, the central challenge is to sustain sufficient levels of long-term surveillance and readiness to enable prompt detection and control of VL case clusters. The strong association with the tolas of scheduled castes and scheduled tribes suggests that efforts should focus on enhanced surveillance in these disadvantaged communities.

## Supplementary Information

**Additional file 1: Figure S1.** Map of villages in Bihar included in the visceral leishmaniasis outbreak investigations. See also Table 1. **Figure S2.** Visceral leishmaniasis case count by year and month of fever onset in three villages of Dumra block, Sitamarhi district. **Figure S3.** Visceral leishmaniasis case count by year and month of fever onset in villages in Bhamath, Binowagram and Pansohi blocks, Purnia district. **Figure S4.** Visceral leishmaniasis case count by year and month of fever onset in Jitwarpur village, Dariyapur block, Saran district. **Figure S5.** Visceral leishmaniasis case count by year and month of fever onset in villages in Rasalpur and Thanwar blocks, Saharsa district. **Figure S6.** Map of houses with (red) and without (green) visceral leishmaniasis cases in Lal Bigha village, Kashichak block, Nawada district. **Figure S7.** Map of houses with (red) and without (green) visceral leishmaniasis cases in Kashichak village, Kashichak block, Nawada district. **Figure S8.** Map of houses with (red) and without (green) visceral leishmaniasis cases in three villages of Dumra block, Sitamarhi district.

**Additional file 2: Table S1.** Categorization of tolas (hamlets within villages) based on percentage of population self-identified as scheduled caste or scheduled tribe (SC/ST), and the cumulative and peak visceral leishmaniasis incidence by tola (expressed as VL cases per 1000 population).

## Data Availability

Due to concerns for participant confidentiality, delinked versions of the datasets used during the current study will be available from the corresponding authors upon reasonable request.

## Abbreviations

CI: Confidence interval
IQR: Interquartile range
IRS: Indoor residual insecticide spraying
KAMIS: Kala-azar Management Information System
KBC: Kala-azar block coordinator
NVBDCP: National Vector-Borne Disease Control Programme
PKDL: Post-kala-azar dermal leishmaniasis
SC/ST: Scheduled caste or scheduled tribe
VL: Visceral leishmaniasis
WHO: World Health Organization
