# Supplementary material for: Visceral leishmaniasis outbreaks in Bihar: community-level investigations in the context of elimination of kala-azar as a public health problem
Source: Parasit Vectors. 2021 Jan 15;14:52. doi: 10.1186/s13071-020-04551-y (PMC7810196; doi:10.1186/s13071-020-04551-y)
Supplement: Supplementary file 1 — Additional file 1: Figure S1. Map of villages in Bihar included in the visceral leishmaniasis outbreak investigations. See also Table 1. Figure S2. Visceral leishmaniasis case count by year and month of fever onset in three villages of Dumra block, Sitamarhi district. Figure S3. Visceral leishmaniasis case count by year and month of fever onset in villages in Bhamath, Binowagram and Pansohi blocks, Purnia district. Figure S4. Visceral leishmaniasis case count by year and month of fever onset in Jitwarpur village, Dariyapur block, Saran district. Figure S5. Visceral leishmaniasis case count by year and month of fever onset in villages in Rasalpur and Thanwar blocks, Saharsa district. Figure S6. Map of houses with (red) and without (green) visceral leishmaniasis cases in Lal Bigha village, Kashichak block, Nawada district. Figure S7. Map of houses with (red) and without (green) visceral leishmaniasis cases in Kashichak village, Kashichak block, Nawada district. Figure S8. Map of houses with (red) and without (green) visceral leishmaniasis cases in three villages of Dumra block, Sitamarhi district. [file 13071_2020_4551_MOESM1_ESM.pptx]

## Slide 1
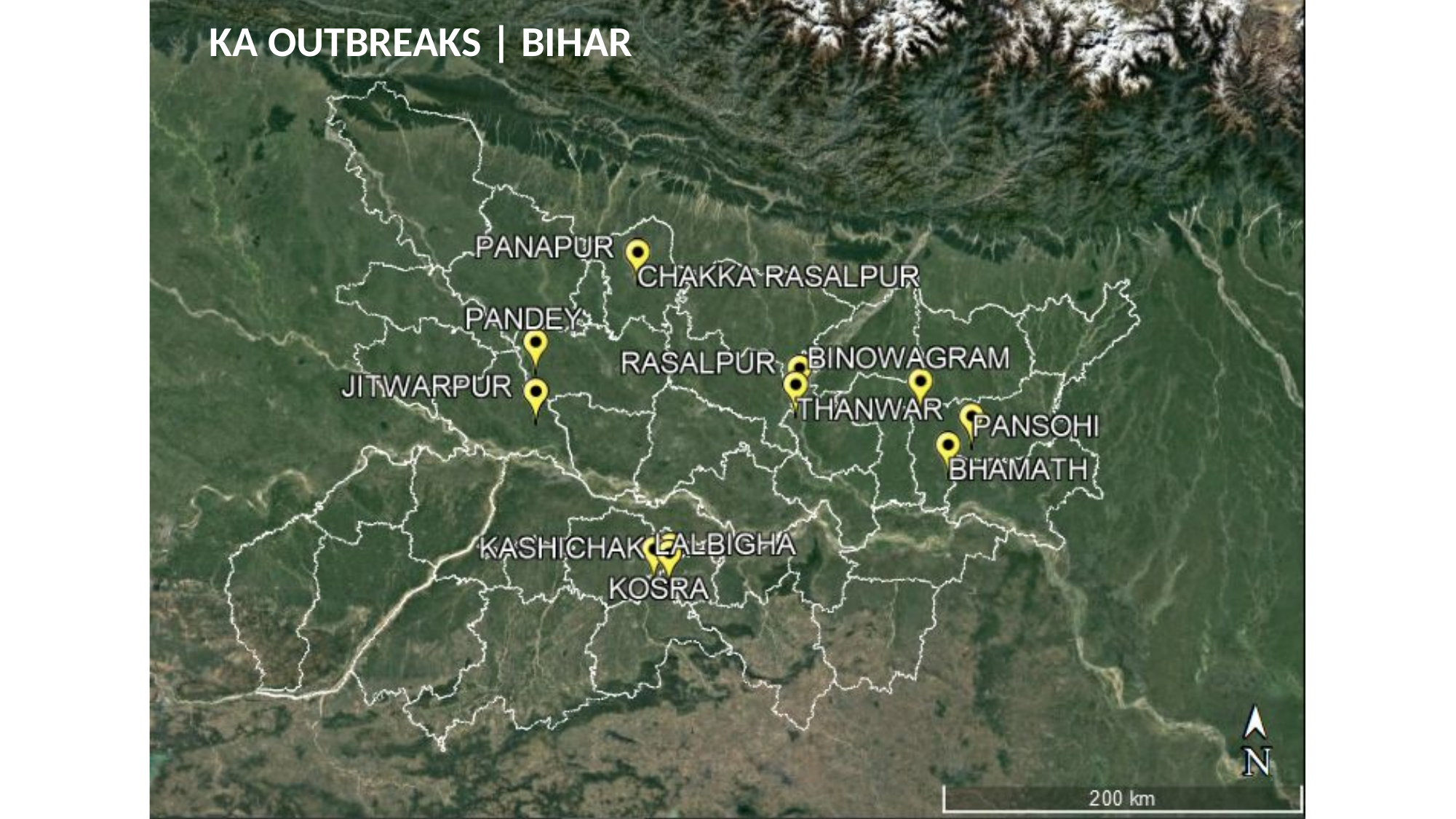

KA OUTBREAKS | BIHAR

## Slide 2
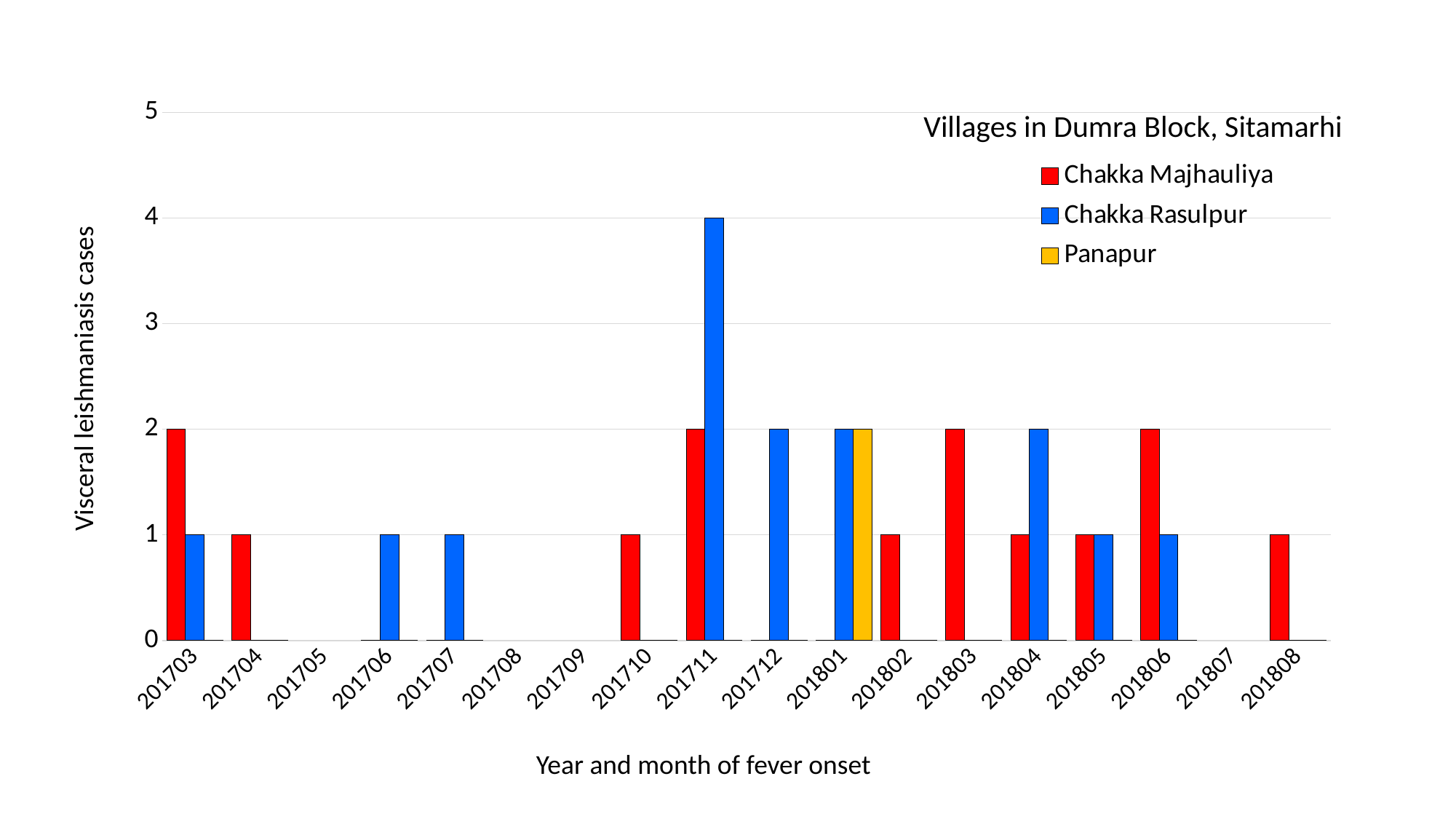

### Chart
| Category | Chakka Majhauliya | Chakka Rasulpur | Panapur |
|---|---|---|---|
| 201703 | 2.0 | 1.0 | 0.0 |
| 201704 | 1.0 | 0.0 | 0.0 |
| 201705 | None | None | None |
| 201706 | 0.0 | 1.0 | 0.0 |
| 201707 | 0.0 | 1.0 | 0.0 |
| 201708 | None | None | None |
| 201709 | None | None | None |
| 201710 | 1.0 | 0.0 | 0.0 |
| 201711 | 2.0 | 4.0 | 0.0 |
| 201712 | 0.0 | 2.0 | 0.0 |
| 201801 | 0.0 | 2.0 | 2.0 |
| 201802 | 1.0 | 0.0 | 0.0 |
| 201803 | 2.0 | 0.0 | 0.0 |
| 201804 | 1.0 | 2.0 | 0.0 |
| 201805 | 1.0 | 1.0 | 0.0 |
| 201806 | 2.0 | 1.0 | 0.0 |
| 201807 | None | None | None |
| 201808 | 1.0 | 0.0 | 0.0 |Villages in Dumra Block, Sitamarhi
Visceral leishmaniasis cases
Year and month of fever onset

## Slide 3
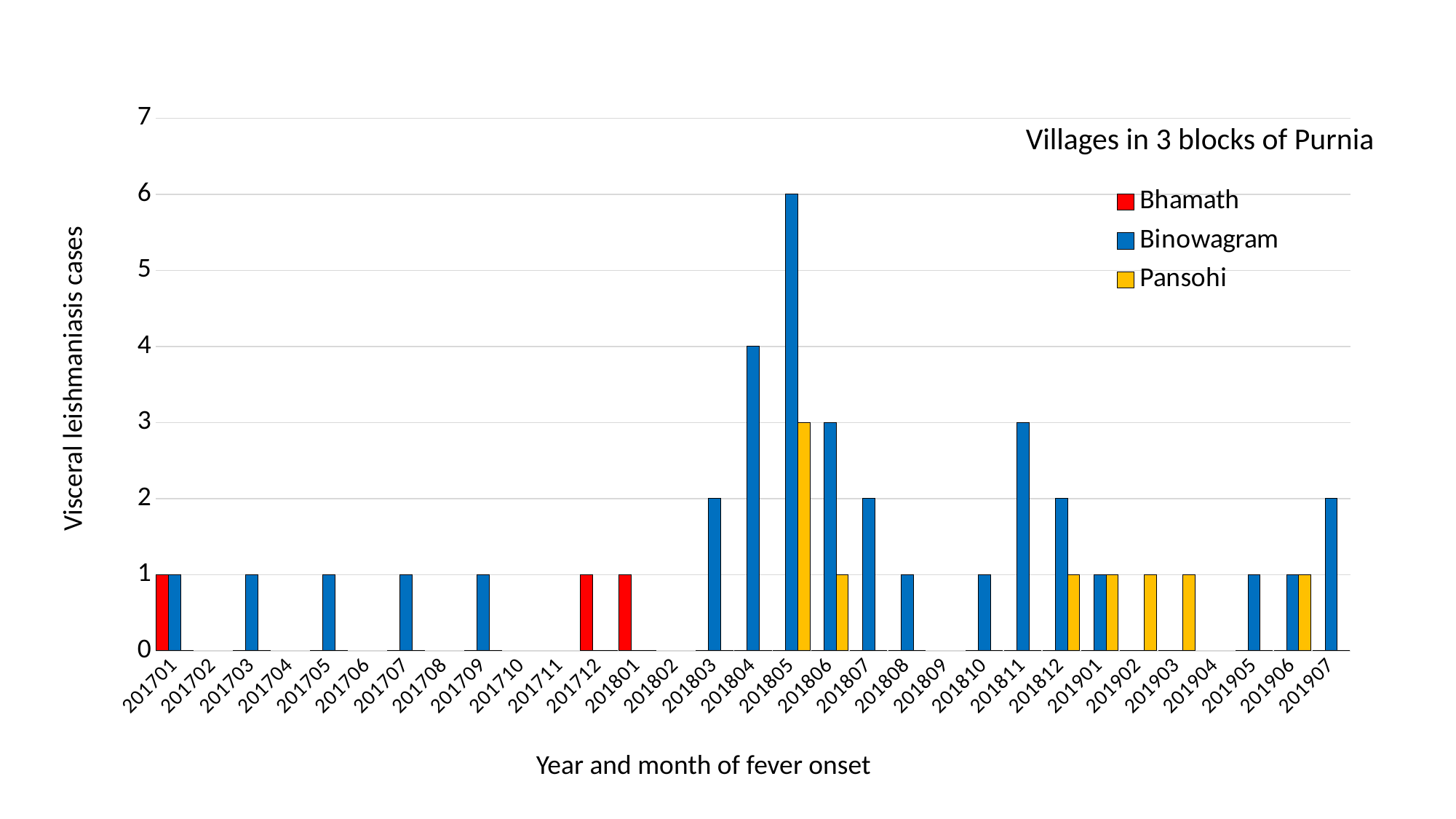

### Chart
| Category | Bhamath | Binowagram | Pansohi |
|---|---|---|---|
| 201701 | 1.0 | 1.0 | 0.0 |
| 201702 | None | None | None |
| 201703 | 0.0 | 1.0 | 0.0 |
| 201704 | None | None | None |
| 201705 | 0.0 | 1.0 | 0.0 |
| 201706 | None | None | None |
| 201707 | 0.0 | 1.0 | 0.0 |
| 201708 | None | None | None |
| 201709 | 0.0 | 1.0 | 0.0 |
| 201710 | None | None | None |
| 201711 | None | None | None |
| 201712 | 1.0 | 0.0 | 0.0 |
| 201801 | 1.0 | 0.0 | 0.0 |
| 201802 | None | None | None |
| 201803 | 0.0 | 2.0 | 0.0 |
| 201804 | 0.0 | 4.0 | 0.0 |
| 201805 | 0.0 | 6.0 | 3.0 |
| 201806 | 0.0 | 3.0 | 1.0 |
| 201807 | 0.0 | 2.0 | 0.0 |
| 201808 | 0.0 | 1.0 | 0.0 |
| 201809 | None | None | None |
| 201810 | 0.0 | 1.0 | 0.0 |
| 201811 | 0.0 | 3.0 | 0.0 |
| 201812 | 0.0 | 2.0 | 1.0 |
| 201901 | 0.0 | 1.0 | 1.0 |
| 201902 | 0.0 | 0.0 | 1.0 |
| 201903 | 0.0 | 0.0 | 1.0 |
| 201904 | None | None | None |
| 201905 | 0.0 | 1.0 | 0.0 |
| 201906 | 0.0 | 1.0 | 1.0 |
| 201907 | 0.0 | 2.0 | 0.0 |Villages in 3 blocks of Purnia
Visceral leishmaniasis cases
Year and month of fever onset

## Slide 4
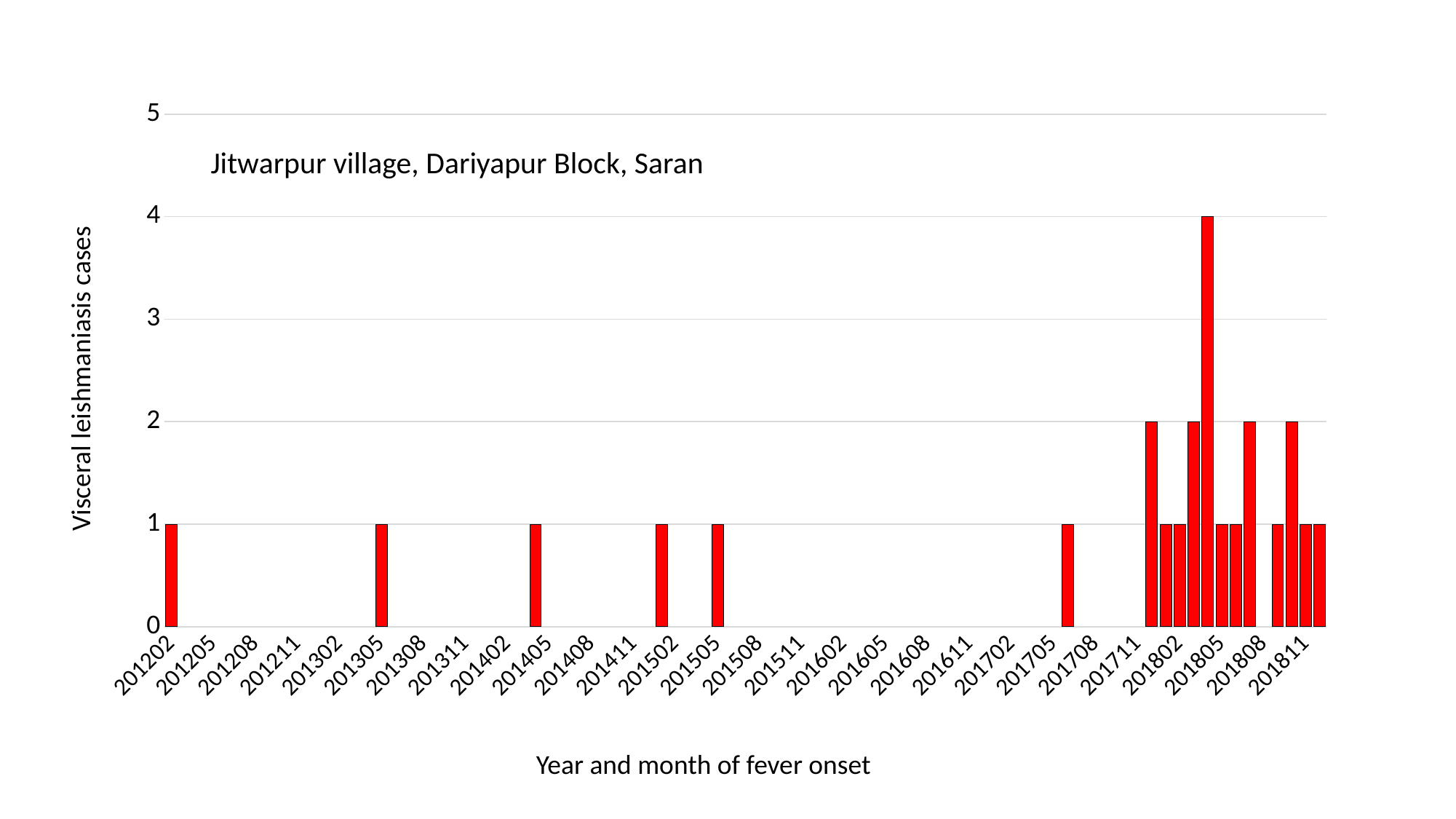

### Chart
| Category | N |
|---|---|
| 201202 | 1.0 |
| 201203 | None |
| 201204 | None |
| 201205 | None |
| 201206 | None |
| 201207 | None |
| 201208 | None |
| 201209 | None |
| 201210 | None |
| 201211 | None |
| 201212 | None |
| 201301 | None |
| 201302 | None |
| 201303 | None |
| 201304 | None |
| 201305 | 1.0 |
| 201306 | None |
| 201307 | None |
| 201308 | None |
| 201309 | None |
| 201310 | None |
| 201311 | None |
| 201312 | None |
| 201401 | None |
| 201402 | None |
| 201403 | None |
| 201404 | 1.0 |
| 201405 | None |
| 201406 | None |
| 201407 | None |
| 201408 | None |
| 201409 | None |
| 201410 | None |
| 201411 | None |
| 201412 | None |
| 201501 | 1.0 |
| 201502 | None |
| 201503 | None |
| 201504 | None |
| 201505 | 1.0 |
| 201506 | None |
| 201507 | None |
| 201508 | None |
| 201509 | None |
| 201510 | None |
| 201511 | None |
| 201512 | None |
| 201601 | None |
| 201602 | None |
| 201603 | None |
| 201604 | None |
| 201605 | None |
| 201606 | None |
| 201607 | None |
| 201608 | None |
| 201609 | None |
| 201610 | None |
| 201611 | None |
| 201612 | None |
| 201701 | None |
| 201702 | None |
| 201703 | None |
| 201704 | None |
| 201705 | None |
| 201706 | 1.0 |
| 201707 | None |
| 201708 | None |
| 201709 | None |
| 201710 | None |
| 201711 | None |
| 201712 | 2.0 |
| 201801 | 1.0 |
| 201802 | 1.0 |
| 201803 | 2.0 |
| 201804 | 4.0 |
| 201805 | 1.0 |
| 201806 | 1.0 |
| 201807 | 2.0 |
| 201808 | None |
| 201809 | 1.0 |
| 201810 | 2.0 |
| 201811 | 1.0 |
| 201812 | 1.0 |Jitwarpur village, Dariyapur Block, Saran
Visceral leishmaniasis cases
Year and month of fever onset

## Slide 5
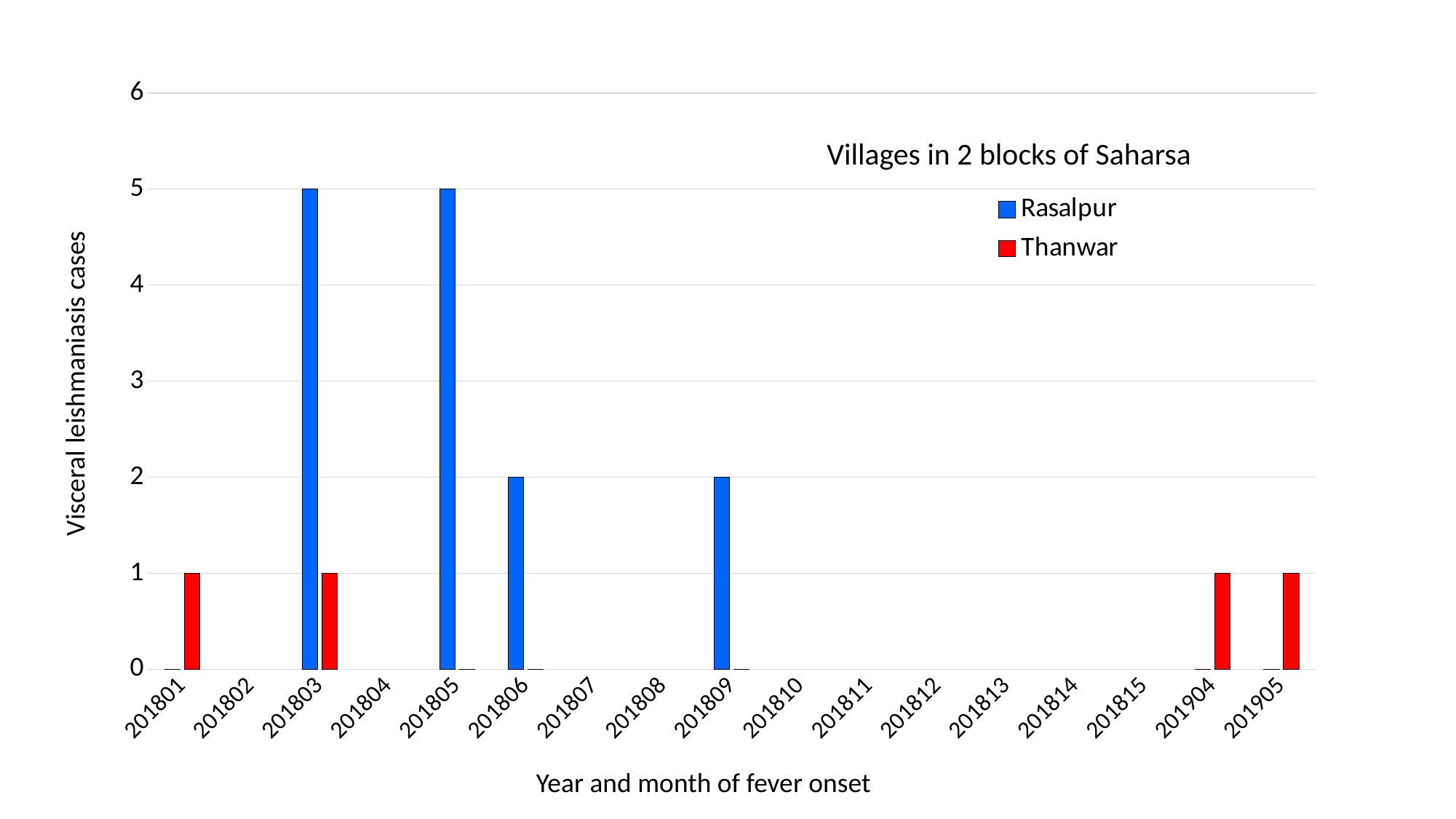

### Chart
| Category | Rasalpur | Thanwar |
|---|---|---|
| 201801 | 0.0 | 1.0 |
| 201802 | None | None |
| 201803 | 5.0 | 1.0 |
| 201804 | None | None |
| 201805 | 5.0 | 0.0 |
| 201806 | 2.0 | 0.0 |
| 201807 | None | None |
| 201808 | None | None |
| 201809 | 2.0 | 0.0 |
| 201810 | None | None |
| 201811 | None | None |
| 201812 | None | None |
| 201813 | None | None |
| 201814 | None | None |
| 201815 | None | None |
| 201904 | 0.0 | 1.0 |
| 201905 | 0.0 | 1.0 |Villages in 2 blocks of Saharsa
Visceral leishmaniasis cases
Year and month of fever onset

## Slide 6
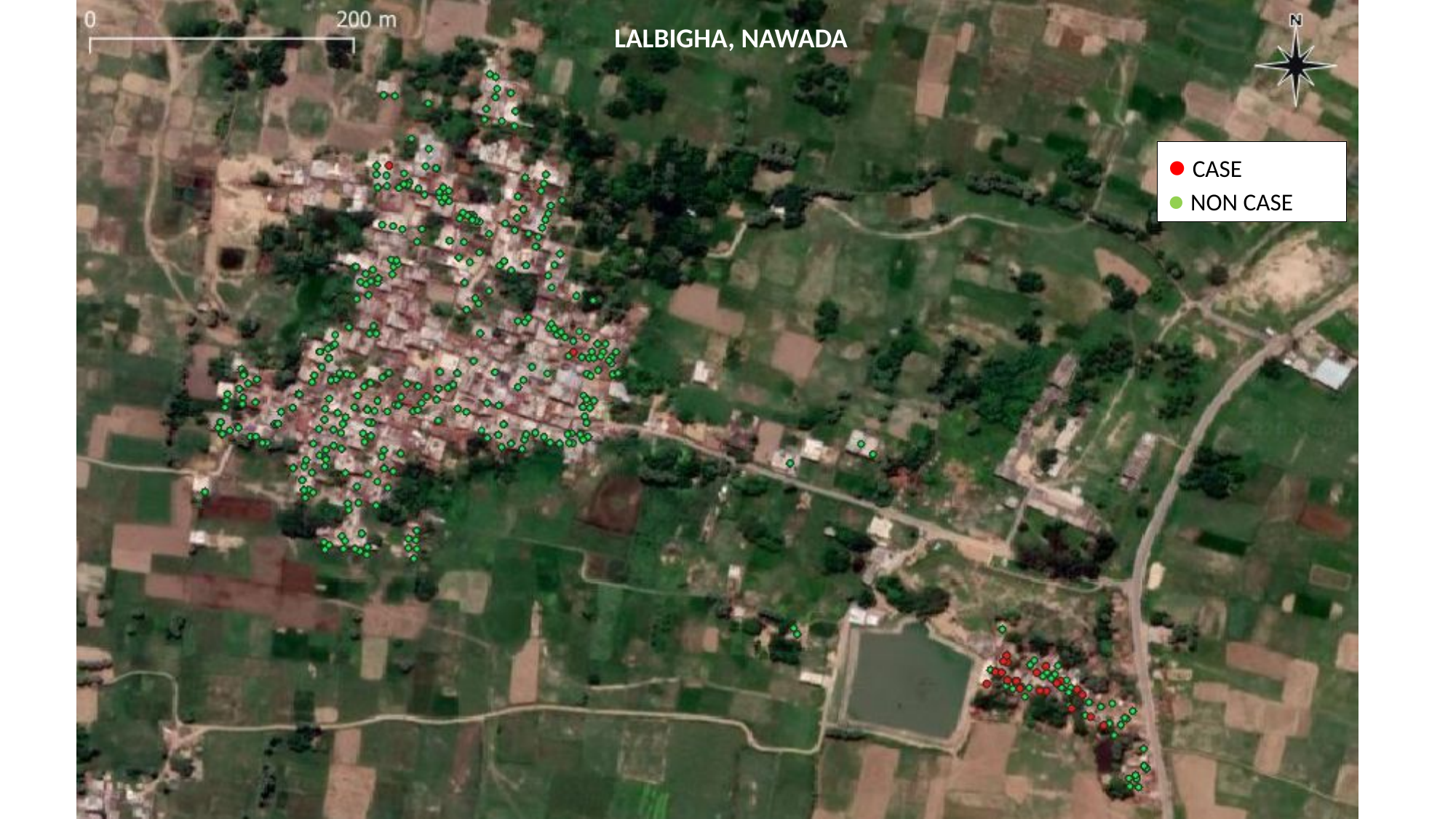

LALBIGHA, NAWADA
● CASE
● NON CASE

## Slide 7
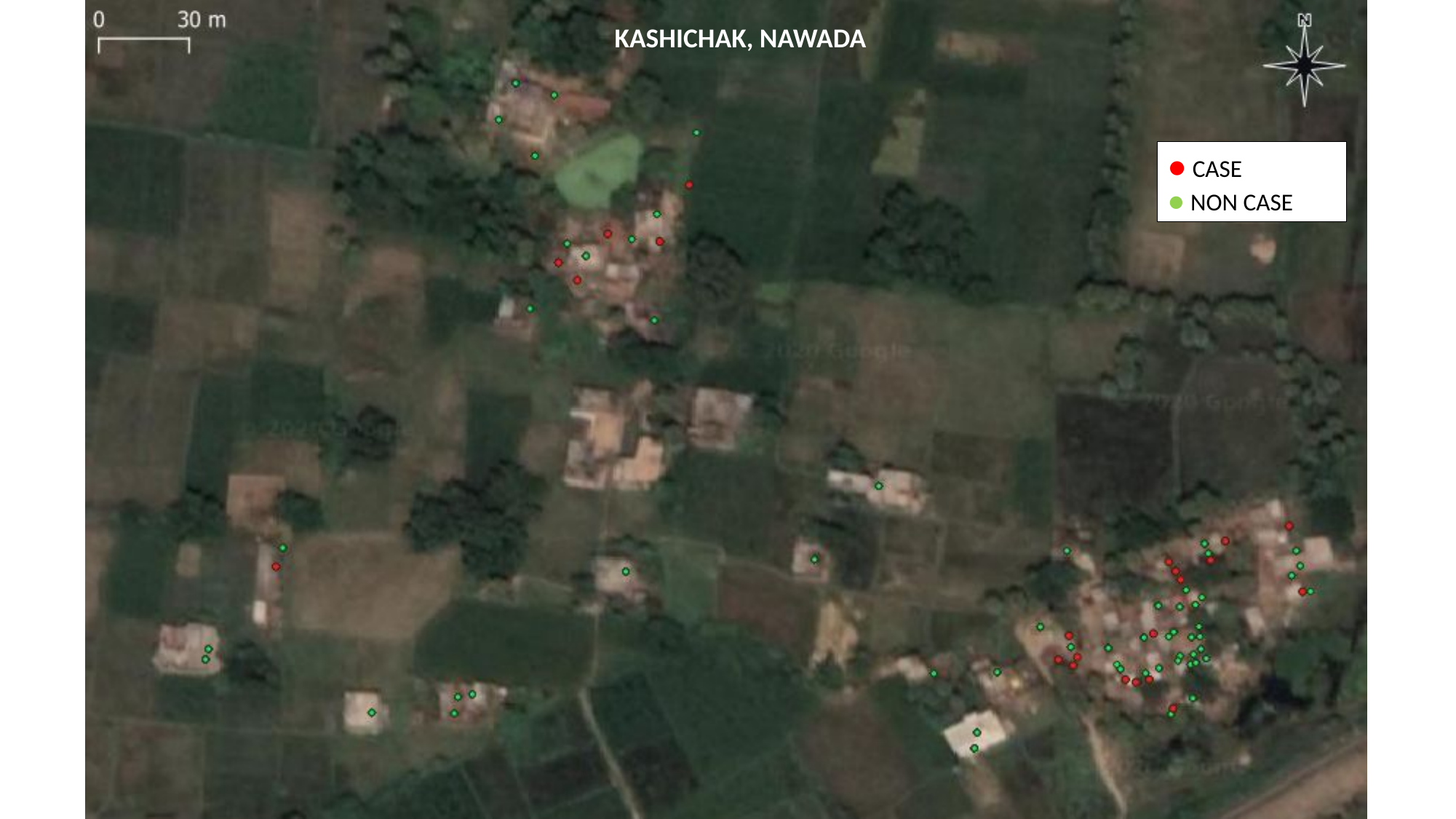

KASHICHAK, NAWADA
● CASE
● NON CASE

## Slide 8
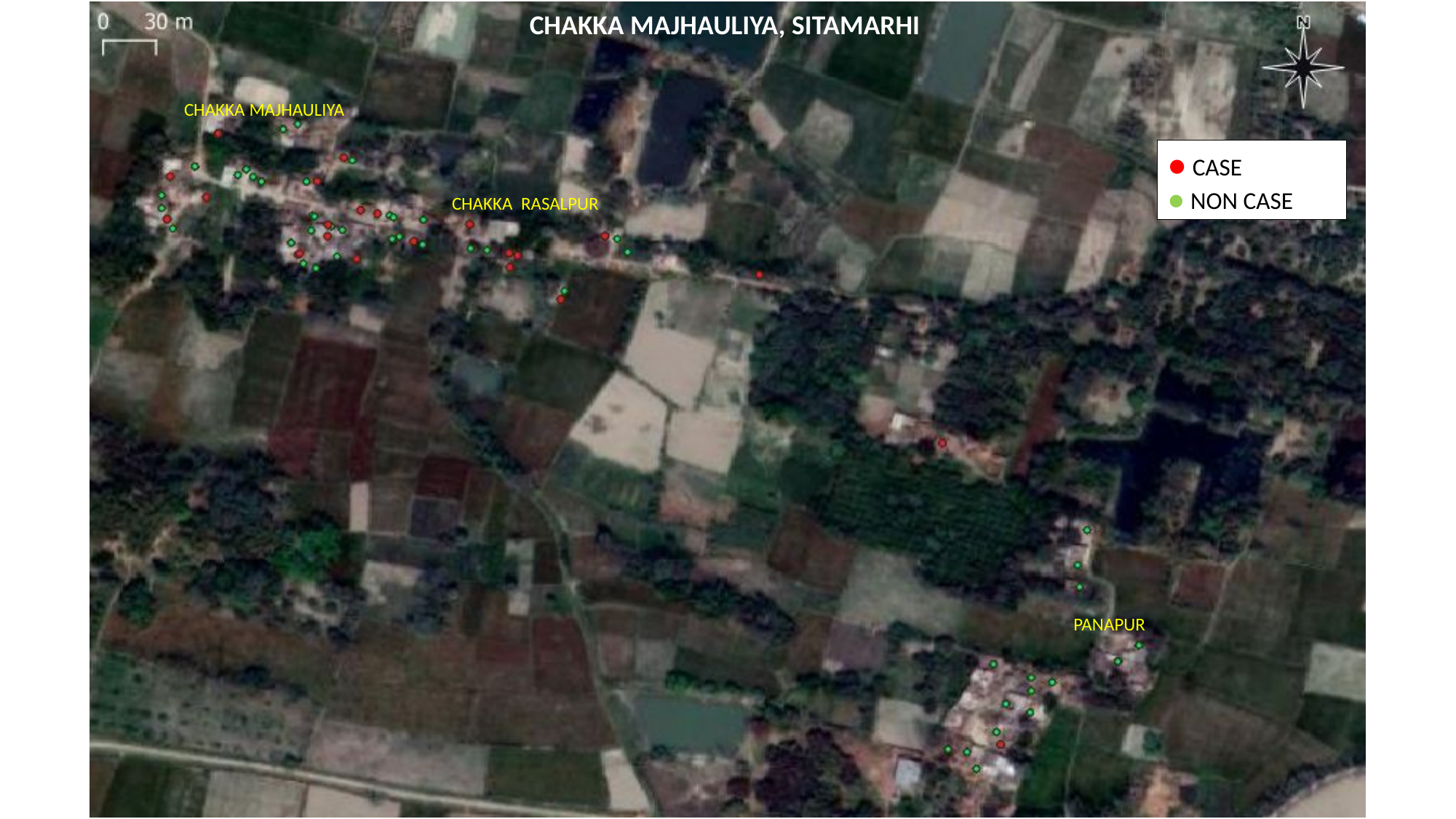

CHAKKA MAJHAULIYA, SITAMARHI
CHAKKA MAJHAULIYA
● CASE
● NON CASE
CHAKKA RASALPUR
PANAPUR
