# Supplementary material for: Visceral leishmaniasis outbreaks in Bihar: community-level investigations in the context of elimination of kala-azar as a public health problem
Source: Parasit Vectors. 2021 Jan 15;14:52. doi: 10.1186/s13071-020-04551-y (PMC7810196; doi:10.1186/s13071-020-04551-y)
Supplement: Supplementary file 2 — Additional file 2: Table S1. Categorization of tolas (hamlets within villages) based on percentage of population self-identified as scheduled caste or scheduled tribe (SC/ST), and the cumulative and peak visceral leishmaniasis incidence by tola (expressed as VL cases per 1000 population). [file 13071_2020_4551_MOESM2_ESM.docx]

Table S1. Percentage of population self-identified as scheduled caste or scheduled tribe (SC/ST) in tolas of investigated villages, and cumulative and peak VL incidence by tola.

|  | **Population of tola** | | | |  | **VL occurrence in tola** | | | |
| --- | --- | --- | --- | --- | --- | --- | --- | --- | --- |
| **Tola** | **Other castes** | **SC/ST** | **Total** | **% SC/ST** |  | **VL cases^2^** | **Peak year** | **Peak N** | **Peak incidence^3^** |
| **SC/ST tolas** |  |  |  |  |  |  |  |  |  |
| Banmankhi 1 | 43 | 260 | 303 | 86% |  | 2 | 2019 | 1 | 3.30 |
| Banmankhi 2 | 234 | 748 | 982 | 76% |  | 32 | 2018 | 23 | 23.42 |
| Bhawanipur 1 | 121 | 378 | 499 | 76% |  | 3 | 2017 | 2 | 4.01 |
| Dariyapur 1 | 31 | 134 | 165 | 81% |  | 1 | 2018 | 1 | 6.06 |
| Dumra 1 | 16 | 223 | 239 | 93% |  | 14 | 2018 | 8 | 33.47 |
| Dumra 2 | 57 | 115 | 172 | 67% |  | 2 | 2018 | 2 | 11.63 |
| Dumra 3 | 7 | 127 | 134 | 95% |  | 15 | 2017 | 9 | 67.16 |
| Krityanand Nagar 1 | 14 | 195 | 209 | 93% |  | 9 | 2018 | 5 | 23.92 |
| Nauhatta 1 | 49 | 302 | 351 | 86% |  | 11 | 2018 | 11 | 31.34 |
| Kashichak 1 | 8 | 300 | 308 | 97% |  | 23 | 2018 | 11 | 35.71 |
| Kashichak 2 | 0 | 342 | 342 | 100% |  | 38 | 2015 | 19 | 55.56 |
| Kashichak 3 | 31 | 172 | 203 | 85% |  | 0 |  |  |  |
| Kashichak 4 | 0 | 124 | 124 | 100% |  | 0 |  |  |  |
| Sheikhpura 1 | 0 | 459 | 459 | 100% |  | 2 | 2018 | 1 | 2.18 |
| Sheikhpura 2 | 8 | 508 | 516 | 98% |  | 65 | 2017 | 39 | 75.58 |
| ***Total*** | ***619*** | ***4387*** | ***5006*** | ***88%*** |  | ***217*** |  |  |  |
| **Other tolas** |  |  |  |  |  |  |  |  |  |
| Banmankhi 2 | 114 | 37 | 151 | 25% |  | 0 |  |  |  |
| Banmankhi 3 | 1333 | 143 | 1476 | 10% |  | 0 |  |  |  |
| Banmankhi 4 | 59 | 0 | 59 | 0% |  | 0 |  |  |  |
| Bhawanipur 2 | 806 | 74 | 880 | 8% |  | 0 |  |  |  |
| Dariyapur 2 | 2395 | 484 | 2879 | 17% |  | 24 | 2018 | 16 | 5.56 |
| Mahishi 1 | 1329 | 428 | 1757 | 24% |  | 4 | 2018 | 2 | 1.14 |
| Nauhatta 2 | 404 | 0 | 404 | 0% |  | 0 |  |  |  |
| Nauhatta 3 | 126 | 0 | 126 | 0% |  | 0 |  |  |  |
| Nauhatta 4 | 381 | 0 | 381 | 0% |  | 2 | 2018 | 2 | 5.25 |
| Nauhatta 5 | 578 | 15 | 593 | 3% |  | 0 |  |  |  |
| Nauhatta 6 | 168 | 3 | 171 | 2% |  | 1 | 2014 | 1 | 5.85 |
| Nauhatta 7 | 354 | 0 | 354 | 0% |  | 1 | 2018 | 1 | 2.82 |
| Paroo 1 | 692 | 86 | 778 | 11% |  | 0 |  |  |  |
| Paroo 2 | 1029 | 21 | 1050 | 2% |  | 7 | 2018 | 7 | 6.67 |
| Paroo 3 | 623 | 214 | 837 | 26% |  | 1 | 2018 | 1 | 1.19 |
| Paroo 4 | 553 | 40 | 593 | 7% |  | 3 | 2018 | 3 | 5.06 |
| Kashichak 5 | 116 | 48 | 164 | 29% |  | 4 | 2018 | 2 | 12.20 |
| Kashichak 6 | 330 | 14 | 344 | 4% |  | 0 |  |  |  |
| Kashichak 7 | 1105 | 102 | 1207 | 8% |  | 1 | 2014 | 1 | 0.83 |
| Sheikhpura 3 | 1243 | 217 | 1460 | 15% |  | 7 | 2017 | 4 | 2.74 |
| ***Total*** | ***13738*** | ***1926*** | ***15664*** | ***12%*** |  | ***55*** |  |  |  |

^1^Median [interquartile range] percent SC/ST: 88% [81,97] ;

^2^Cumulative number of VL cases 2012-2019

^3^Peak incidence expressed as VL cases per 1000 population per year (based on year of fever onset)

^4^Median [IQR] percent SC/ST: 7.5% [0,16].
